# Comparison of the Achilles tendon moment arms determined using the tendon excursion and three‐dimensional methods

**DOI:** 10.14814/phy2.12967

**Published:** 2016-10-05

**Authors:** Satoru Hashizume, Atsuki Fukutani, Kazuki Kusumoto, Toshiyuki Kurihara, Toshio Yanagiya

**Affiliations:** ^1^ Human Informatics Research InstituteNational Institute of Advanced Industrial Science and TechnologyTokyoJapan; ^2^ Faculty of Health and Sports ScienceJuntendo UniversityChibaJapan; ^3^ Japan Society for the Promotion of ScienceTokyoJapan; ^4^ The Research Organization of Science and TechnologyRitsumeikan UniversityShigaJapan; ^5^ Faculty of Science and Industrial TechnologyKurashiki University of Science and the ArtsOkayamaJapan; ^6^ Faculty of Sport and Health ScienceRitsumeikan UniversityShigaJapan

**Keywords:** Magnetic resonance imaging, musculoskeletal model, triceps surae, ultrasonography

## Abstract

The moment arm of muscle‐tendon force is a key parameter for calculating muscle and tendon properties. The tendon excursion method was used for determining the Achilles tendon moment arm (ATMA). However, the accuracy of this method remains unclear. This study aimed to investigate the magnitude of error introduced in determining the ATMA using the tendon excursion method by comparing it with the reference three‐dimensional (3D) method. The tendon excursion method determined the ATMA as the ratio between the Achilles tendon displacement during foot rotation from 15° of dorsiflexion to 15° of plantarflexion and the joint rotation angle. A series of foot images was obtained at 15° of dorsiflexion, the neutral position, and 15° of plantarflexion. The 3D value of the ATMA was determined as the shortest distance between the talocrural joint axis and the line of action of the Achilles tendon force. The ATMA determined by the tendon excursion method was smaller by 3.8 mm than that determined using the 3D method. This error may be explained mainly by the length change in the Achilles tendon due to the change in the force applied to it, as passive plantarflexion torque was different by 11 Nm between 15° of dorsiflexion and 15° of plantarflexion. Furthermore, the ATMAs determined using the 3D and tendon excursion methods were significantly correlated but the coefficient of determination was not large (*R* ^2^ = 0.352). This result suggests that the tendon excursion method may not be feasible to evaluate the individual variability of the ATMA.

## Introduction

In inverse dynamics, the moment arm of muscle‐tendon force, defined as the shortest distance between the joint rotation axis and the line of action of the muscle‐tendon force, is used to estimate the muscle‐tendon force for in vivo positions (Spoor and van Leeuwen 1992; Hashizume et al. 2012, 2014; Sheehan 2012). The moment arm of muscle‐tendon force is one of the essential parameters for musculoskeletal modeling (Nisell et al. 1986; Delp et al. 1990; Winter and Challis 2010), and for calculating the mechanical properties of the muscles (Fukunaga et al. 1996; Reeves et al. 2004; Morse et al. 2008) and tendons (Maganaris and Paul 1999; Kubo et al. 2000; Arampatzis et al. 2010). An accurate determination of the moment arm of the muscle‐tendon force is required for these studies. Previously, some methods have been proposed for accurate determination of the moment arm of the muscle‐tendon force.

Two‐dimensional (2D) approaches, such as center of rotation (Rugg et al. 1990; Fath et al. 2010, 2013) and tendon excursion methods (Lee and Piazza 2009; Fath et al. 2010, 2013), have often been used for in vivo determination of the moment arm of muscle‐tendon forces. The center of rotation method determines the moment arm as the shortest distance between the joint center of rotation and the line of action of muscle‐tendon force in the selected anatomical plane. This method assumes that the plane is orthogonal to the joint rotation axis. However, previous studies invalidated this assumption (Sheehan 2007; Leitch et al. 2010). In fact, a previous study (Hashizume et al. 2012) reported that the center of rotation method overestimates the Achilles tendon moment arm (ATMA) compared to the three‐dimensional (3D) method owing to the invalid assumption of the center of rotation method.

The tendon excursion method determines the moment arm of muscle‐tendon forces as the ratio between tendon displacement observed during a given amount of joint rotation and the corresponding joint rotation angle. This method assumes that no work is done on the tendon during joint rotation (the principle of virtual work) (Amis et al. 1979; An et al. 1983; Olszewski et al. 2015). This assumption, however, was also invalidated by previous study (Fath et al. 2010). The slack length, defined as the length beyond which a tendon begins to develop passive force (Hug et al. 2013), increased by plantarflexion in the Achilles tendon (Muraoka et al. 2002, 2004). The increased Achilles tendon length beyond the slack length during foot rotation induces increase in passive force and passive plantarflexion torque. This induces underestimation of the measured tendon displacement, resulting in underestimation of the ATMA (Fath et al. 2010). However, the magnitude of error introduced by the tendon excursion method for determining the ATMA remains unclear.

The ATMA determined using the center of rotation method overestimates the ATMA by about 22% compared to the 3D method (Hashizume et al. 2012). Furthermore, a previous study (Fath et al. 2010) reported that the ATMA determined using the center of rotation method was greater by 31% than that determined using the tendon excursion method (Fath et al. 2010). On the basis of these two studies, we hypothesized that the tendon excursion method may underestimate the ATMA by about 7% compared with the 3D method. Although the 3D method (Hashizume et al. 2012, 2014; Sheehan 2012; Clarke et al. 2015) can overcome the limitations of 2D approaches, this method is not always feasible owing to long scan time and high cost of data collection. Furthermore, the center of rotation method also requires MRI for data collection, whereas data collection for the tendon excursion method can be performed by ultrasonography, which is more feasible. If the magnitude of error introduced by the tendon excursion method is not large, it may be a convenient method for determining the ATMA. The purpose of this study, therefore, was to investigate the error magnitude introduced by the tendon excursion method in determining the ATMA by comparison with the 3D method.

## Materials and Methods

### Subjects

Twelve healthy men (age: 20.2 ± 1.1 years old, body height: 1.73 ± 0.05 m, body mass: 60.7 ± 4.8 kg) participated. Before the experiment, written informed consent was obtained from each subject. The Ethics Committee on Human Research of the Ritsumeikan University approved the experimental protocol of this study.

### Determination of the ATMA using the tendon excursion method

**Figure 1:**
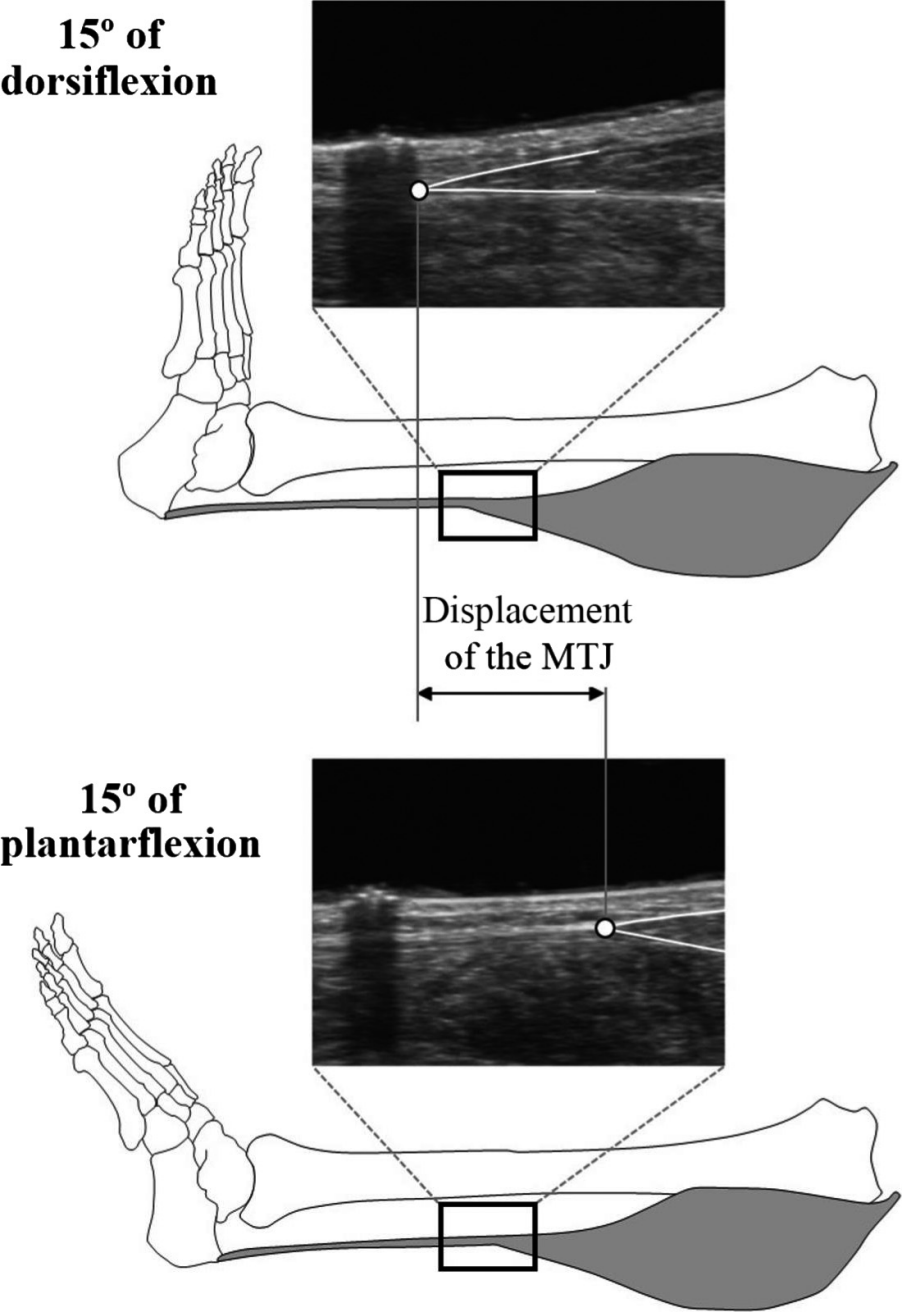
Measurement of displacement of medial gastrocnemius muscle‐tendon junction. The open circle on ultrasonographic image represents the muscle‐tendon junction.

The tendon excursion method was conducted to determine the ATMA. A dynamometer (Biodex; SAKAImed, Tokyo, Japan) was used to stabilize the foot and the lower leg at given ankle joint angles. Each subject was asked to lie in a prone position with knees fully extended on a dynamometer bed. The mid‐point of the line connecting the medial and lateral malleoli of the right foot was carefully aligned with the dynamometer's rotation axis. Next, the right foot and right lower leg were fixed to the foot plate and the dynamometer bed, respectively, using nonelastic belts. The position of the medial gastrocnemius muscle‐tendon junction was recorded at 15° of dorsiflexion and 15° of plantarflexion using an ultrasound apparatus (SSD‐3500; Aloka, Tokyo, Japan) with 6 cm linear array probe (scanning frequency of 7.5 MHz), and its displacement was then calculated (Fig. 1). Before measurement, the ultrasound apparatus’ imaging plane was visually aligned to the motion plane of the medial gastrocnemius muscle‐tendon junction during passive foot rotation. The ATMA was determined as the ratio of measured medial gastrocnemius muscle‐tendon junction displacement and angular displacement of the foot. Furthermore, passive plantarflexion torque was recorded for both ankle joint angles using a dynamometer. The tendon excursion method assumes that no work is done on the tendon during joint rotation (the principle of virtual work) (Amis et al. 1979; An et al. 1983; Olszewski et al. 2015). Therefore, we calculated the difference in passive plantarflexion torque between 15° of dorsiflexion and 15° of plantarflexion to confirm whether work done during joint rotation was zero or not.

### Determination of the ATMA using the 3D method

**Figure 2:**
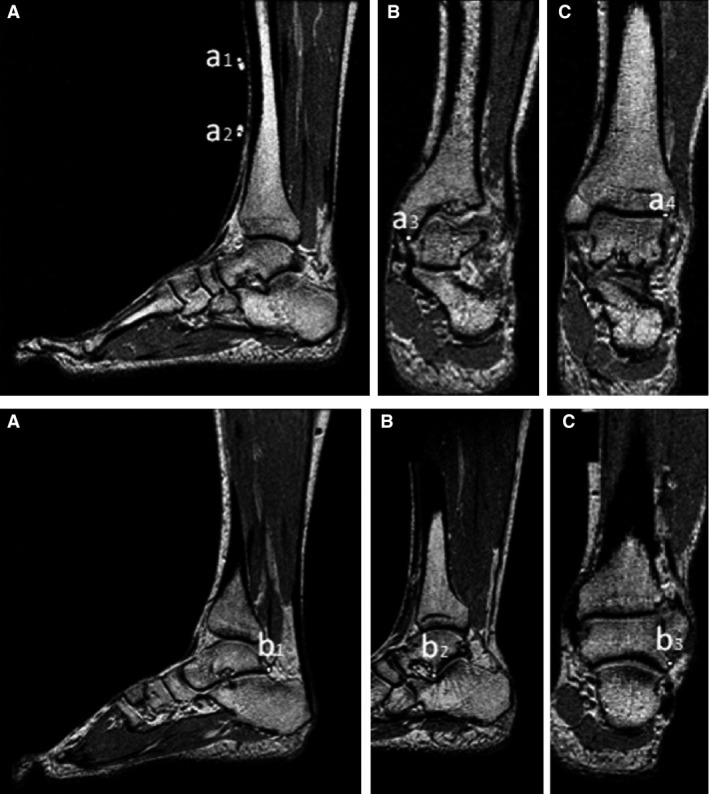
MR images of the right foot for determining the bony landmarks of the tibia (a1–a4) and the talus (b1–b3) used for defining the local coordinate system embedded to each bone. The 3D coordinates of the bony landmarks were obtained using a computer‐aided diagnosis system (PLUTO, Nagoya University). This figure is cited from Figures 2 and 3 of “In vivo determination of the Achilles tendon moment arm in three‐dimensions (Hashizume et al. 2012)” in the *Journal of Biomechanics* 45 (2).

The 3D method described previously (Hashizume et al. 2012, 2014) was used to determine the ATMA reference value. Each subject was asked to lie in a supine position with knees fully extended on an MRI scanner bed (Signa HDxt, 1.5T; GE Medical Systems, with an eight channel body array coil, Waukesha, WI). The right foot was fixed to the foot plate of a custom‐made apparatus constructed of MRI‐compatible materials at 15° of dorsiflexion, the neutral position, and 15° of plantarflexion using nonelastic belts. A series of sagittal MR images of the right foot were obtained for the three foot positions with the following scan parameters: spoiled gradient recalled acquisition in the steady state, 3.2 msec time to echo, 6.6 msec repetition time, 1.5 mm slice thickness, 300 × 300 mm field of view, and 512 × 512 pixel matrix. Voxel resolution was 0.58 × 0.58 × 1.5 mm. The bony landmarks of each bone (Fig. 2) were visually identified and the 3D coordinate of each landmark was corrected with a computer‐aided diagnosis system (PLUTO, Nagoya University, Aichi, Japan). The right‐handed orthogonal coordinate systems embedded to the tibia and the talus were defined using 3D coordinates of the bony landmarks of each bone. The finite helical axis was then computed as follows:

$$\Delta {A_{\mathrm{tibia}/\mathrm{talus}}}^{\mathrm{neutral}\pm 15}={\left({A_{\mathrm{tibia}/\mathrm{talus}}}^{\mathrm{neutral}-15}\right)}^{T}{A_{\mathrm{tibia}/\mathrm{talus}}}^{\mathrm{neutral}+15}$$

The rotation matrix (*ΔA*) represents the angular displacement of the tibia‐embedded coordinate system relative to the talus‐embedded coordinate system when the foot rotates from 15° of dorsiflexion to 15° of plantarflexion.

$$\begin{array}{cc} \theta = & \;\cos^{-1}\frac{1}{2}\left(\right.\Delta {A_{\mathrm{tibia}/\mathrm{talus}}}^{\mathrm{neutral}\pm 15}11+\Delta {A_{\mathrm{tibia}/\mathrm{talus}}}^{\mathrm{neutral}\pm 15}22\\ & +\Delta {A_{\mathrm{tibia}/\mathrm{talus}}}^{\mathrm{neutral}\pm 15}33-1\left.\right) \end{array}$$

$$\vec{u}=\frac{1}{2\sin\theta }\left[\begin{array}{c} \Delta {A_{\text{tibia}/\text{talus}}}^{\text{neutral}\pm 15}32-\Delta {A_{\text{tibia}/\text{talus}}}^{\text{neutral}\pm 15}23\\ \Delta {A_{\text{tibia}/\text{talus}}}^{\text{neutral}\pm 15}13-\Delta {A_{\text{tibia}/\text{talus}}}^{\text{neutral}\pm 15}31\\ \Delta {A_{\text{tibia}/\text{talus}}}^{\text{neutral}\pm 15}21-\Delta {A_{\text{tibia}/\text{talus}}}^{\text{neutral}\pm 15}12 \end{array}\right]$$

The elements of *ΔA* were used to determine the rotation angle of the talocrural joint (*θ*) and the finite helical axis ($\vec{u}$) (Spoor and Veldpaus 1980). The calculated finite helical axis was used to represent the position and orientation of the talocrural joint axis for the neutral position. By using the images collected at the neutral position, the line of action of the Achilles tendon force was determined as the straight line through the centers of the cross‐sectional areas of the Achilles tendon at the proximal insertion site to the soleus and the distal insertion site to the calcaneus (Hashizume et al. 2012, 2014; Sheehan 2012; Clarke et al. 2015). The line of action was projected onto the orthogonal plane of the talocrural joint axis, and the shortest distance between that projected line and the talocrural joint axis was calculated as the ATMA for the neutral position.

### Statistical analysis

Data reduction reliability was confirmed using a single dataset. Data reduction was performed twice, and the coefficients of variance (CVs) and intraclass correlation coefficients (ICCs) were calculated for each method. The CVs were 2.3 ± 1.0% for the 3D method and 1.3 ± 1.5% for the tendon excursion method, and the ICCs were 0.93 and 0.97, respectively. These calculated CVs and ICCs were similar to the corresponding values reported previously (Lee et al. 2008; Fath et al. 2010; Hashizume et al. 2012). The mean values of the two datasets were used for subsequent statistical analysis. The normality of each parameter was confirmed by the Kolmogorov–Smirnov test (*P* > 0.05). A paired *t*‐test was conducted to test for differences in the ATMAs determined using the two methods. Effect size was calculated as Cohen's *d*. The Bland–Altman plot was used to examine proportional bias in the ATMAs determined using the 3D and tendon excursion methods. The difference in the passive plantarflexion torque between 15° of dorsiflexion and 15° of plantarflexion was compared with zero using a one‐sample *t*‐test. Pearson's product‐moment correlation coefficient was used to examine the relationship between the ATMAs determined using the two methods. Statistical significance was set at *P* ≤ 0.05. Statistical power was calculated as 1 − *β*. Statistical analysis was executed using statistical software (IBM SPSS Statistics Version 22.0; IBM Corporation, Armonk, NY).

## Results

**Figure 3:**
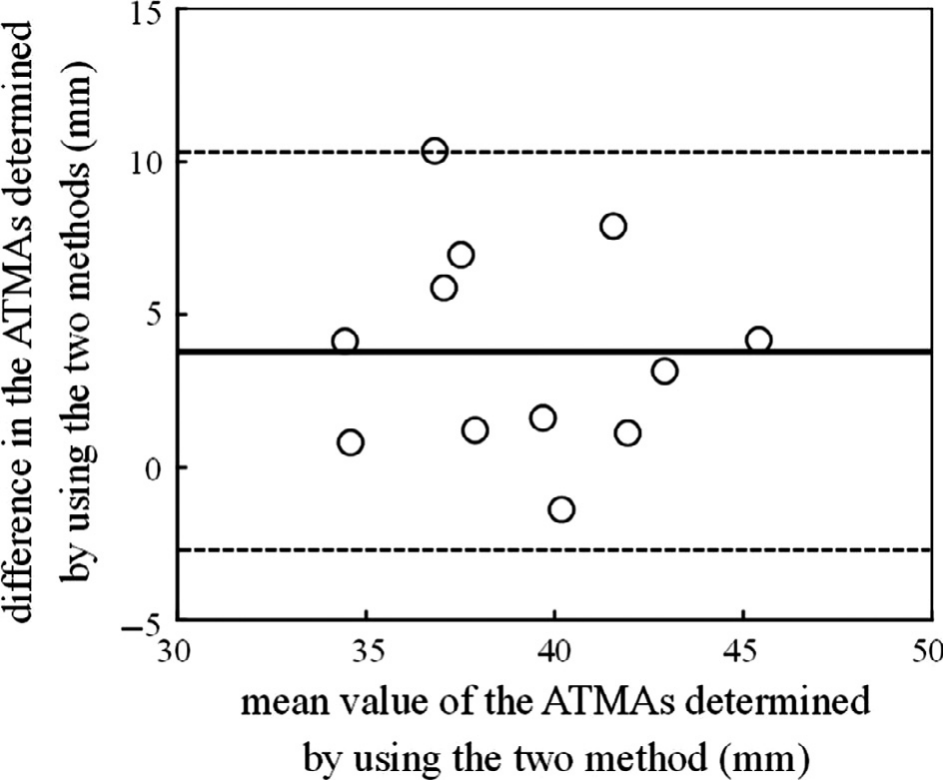
The Bland–Altman plots of the Achilles tendon moment arm (ATMAs) determined using the 3D and tendon excursion methods. The solid line represents the mean difference in the ATMAs determined using the 3D and tendon excursion methods. The dotted lines represent the 1.96 SD range.

**Figure 4:**
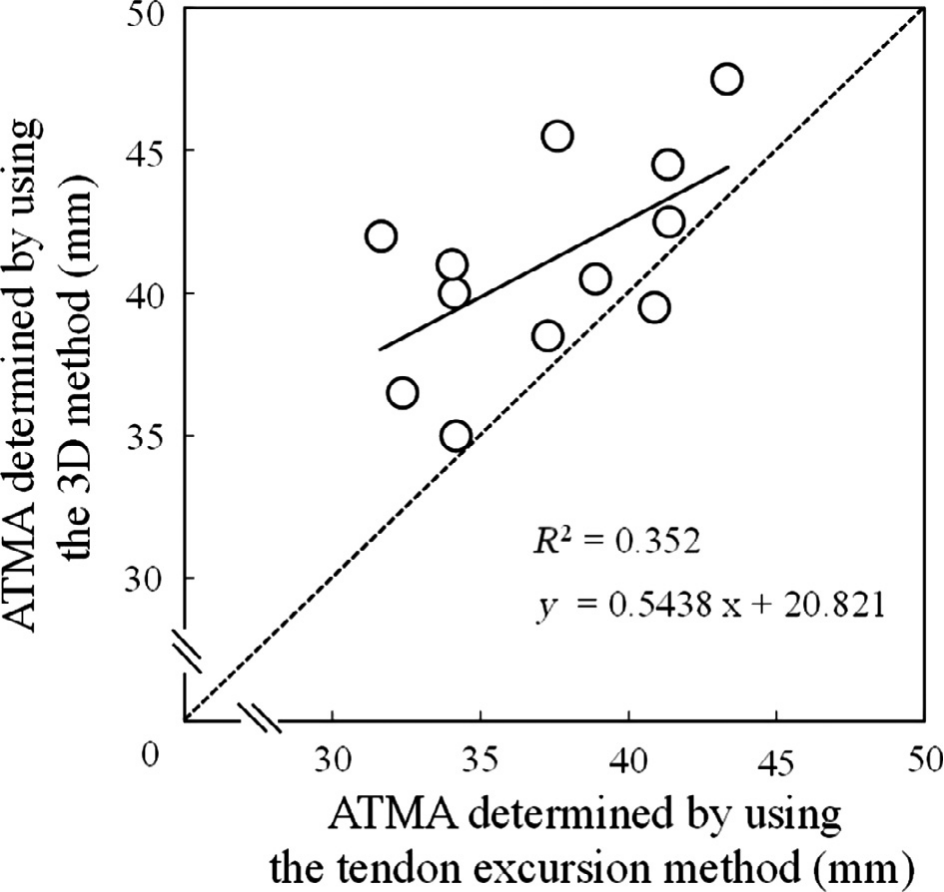
Association between the Achilles tendon moment arm (ATMAs) determined using the 3D and tendon excursion methods. The dotted line represents the identity line (*X* axis = *Y* axis).

Descriptive data were presented as means ± standard deviations (SDs). The ATMA determined using the tendon excursion method (37 ± 3.8 mm) was significantly smaller by 3.8 ± 3.3 mm (9.7%) than that determined using the 3D method (41 ± 3.5 mm) (*t* = 3.867, *P* < 0.01, effect size = 1.05, power = 0.911). The Bland–Altman plots revealed no significant proportional bias in the ATMAs determined using the 3D and tendon excursion methods (Fig. 3). A significant correlation was found between the ATMAs determined using the 3D and tendon excursion methods (*P* = 0.05, *R* ^2^ = 0.352) (Fig. 4). The difference in the passive plantarflexion torque between 15° of dorsiflexion and 15° of plantarflexion (11 ± 1.7 Nm) was significantly greater than zero (*P* < 0.001).

## Discussion

This study tested the hypothesis that the tendon excursion method underestimates the ATMA. The present result revealed that the ATMA determined using tendon excursion method was, on average, 3.8 mm smaller than that determined using the 3D method. This result clearly supports our hypothesis.

The major limitation of this study was that the ATMA was determined in one foot position for each method. The passive plantarflexion torque and Achilles tendon slack length varied among ankle joint angles (Muraoka et al. 2002, 2004; Fath et al. 2010; Hug et al. 2013), suggesting that the error introduced by the tendon excursion method also varies. Previous studies determined the ATMAs for various ankle joint angles using the 3D (Hashizume et al. 2012) and tendon excursion (Fath et al. 2010) methods. The ATMA determined using the 3D method was increased by 6 mm from 10° of dorsiflexion (35 mm) to the neutral position (41 mm) and similar from the neutral position to 10° and 20° of plantarflexion (41 mm and 40 mm, respectively) (Hashizume et al. 2012). Meanwhile, the ATMA determined using the tendon excursion method was increased gradually from 15° of dorsiflexion (34.6 mm) to 30° of plantarflexion (36.6 mm) (Fath et al. 2010). These data suggest that the error introduced by the tendon excursion method varies slightly among ankle joint angles and that the tendon excursion method consistently underestimates the ATMA for various ankle joint angles. The limitation of this study, therefore, may have had little effect on its main finding. Some previous studies (Rugg et al. 1990; Sheehan 2007; Fath et al. 2010; Hashizume et al. 2012) showed that the ATMA varies among individuals, and therefore direct comparison was required to represent methodology‐dependent difference in the ATMAs. This study confirmed the error introduced by the tendon excursion method for the ATMA determination without individual variability by direct comparison of the ATMAs determined using the 3D and tendon excursion methods.

The Bland–Altman plot revealed no proportional bias in the ATMAs determined by the 3D and tendon excursion methods, and a significantly smaller ATMA was found (by 9.7%) for the tendon excursion method compared with the 3D method. The error introduced by the tendon excursion method was smaller than that introduced by the center of rotation method (22%) reported previously (Hashizume et al. 2012). These suggest that the tendon excursion method introduces error in determining the ATMA, but this error is smaller than that introduced by the center of rotation method. The 9.7% error observed in this study may be due to the violated assumption of the tendon excursion method. In this study, the displacement of the medial gastrocnemius muscle‐tendon junction was recorded during passive foot rotation between 15° of dorsiflexion and 15° of plantarflexion. Because Achilles tendon slack length was observed at more than 20° of plantarflexion (Muraoka et al. 2002), the influence of the slack is thought to be small. Meanwhile, the difference in passive plantarflexion torque between 15° of dorsiflexion and 15° of plantarflexion was 11 ± 1.7 Nm in this study. The force applied on the Achilles tendon also changed, and this induced the tendon's length change. This induced underestimation of measured tendon displacement, resulting in underestimation of the ATMA (Fath et al. 2010). The error introduced by the tendon excursion method, therefore, may be explained mainly by the Achilles tendon length change due to the change in applied force during foot rotation. These results suggest that researchers should take care to use the ATMA determined using the tendon excursion method for calculating the muscle and tendon properties.

The ATMAs determined using the 3D and tendon excursion methods were significantly correlated (*P* = 0.05), but the coefficient of determination was not large (*R* ^2^ = 0.352). This suggests that the magnitude of error of the ATMA determined using the tendon excursion method varies among individuals. The individual variability of this error is likely induced by individual variabilities of the force applied to the Achilles tendon and its stiffness. The difference in passive plantarflexion torque ranged from 8 to 13 Nm among individuals in this study, suggesting that the force applied to the Achilles tendon also varied among individuals. Furthermore, previous studies (Kubo et al. 2000; Arampatzis et al. 2010) reported that Achilles tendon stiffness varies among individuals. These may be reasons for the weak relationship between the ATMAs determined using the 3D and tendon excursion methods. Therefore, it may not be easy to evaluate the individual variability of the ATMA using the tendon excursion method. This problem may be resolved by developing an estimation algorithm of the accurate individual ATMA using the tendon excursion method and measurement of the passive plantarflexion torque.

## Conclusions

In summary, we investigated the magnitude of error introduced by the tendon excursion method in determining the ATMA. In the results, the ATMA determined using the tendon excursion method was smaller by 3.8 mm than that determined using the 3D method. This error can be explained mainly by the Achilles tendon length change due to the change in the force applied to it because passive plantarflexion torque differed by 11 Nm between 15° of dorsiflexion and 15° of plantarflexion. Furthermore, the ATMAs determined using the 3D and tendon excursion methods were significantly correlated but coefficient of determination was not large (*R* ^2^ = 0.352). This result suggests that the tendon excursion method may not be feasible to evaluate the individual variability of the ATMA.

## Conflict of Interest

The authors declare that there are no conflicts of interest.
